# Smoldering multiple myeloma current treatment algorithms

**DOI:** 10.1038/s41408-022-00719-0

**Published:** 2022-09-05

**Authors:** S. Vincent Rajkumar, Shaji Kumar, Sagar Lonial, Maria Victoria Mateos

**Affiliations:** 1 Division of Hematology, Mayo Clinic, Rochester, MN USA; 2 Department of Hematology and Medical Oncology, Emory University School of Medicine Atlanta, Atlanta, GA USA; 3 Department of Hematology, and Center for Cancer Research, University Hospital of Salamanca, Salamanca, Spain

**Keywords:** Haematological cancer, Chemotherapy

## Abstract

Smoldering multiple myeloma (SMM) is an asymptomatic condition that occupies a space between monoclonal gammopathy of undetermined significance (MGUS) and multiple myeloma (MM) along the spectrum of clonal plasma cell proliferative disorders. It is not a biologic intermediate stage between MGUS and MM, but rather represents a heterogeneous clinically defined condition in which some patients (approximately two-thirds) have MGUS (pre-malignancy), and some (approximately one-third) have MM (biologic malignancy). Unfortunately, no single pathologic or molecular feature can reliably distinguish these two groups of patients. For purposes of practice and clinical trials, specific risk factors are used to identify patients with SMM in whom malignant transformation has already likely occurred (high risk SMM). Patients with newly diagnosed high risk SMM should be offered therapy with lenalidomide or lenalidomide plus dexamethasone (Rd) for 2 years, or enrollment in clinical trials. Patients with low risk SMM should be observed without therapy every 3–4 months.

## Introduction

The diagnostic criteria, staging system, response criteria, and management of multiple myeloma (MM) have evolved significantly in the last decade [1]. Almost all patients with MM evolve from a pre-malignant stage termed monoclonal gammopathy of undetermined significance (MGUS) [2, 3]. MGUS is present in approximately 5% of the population above the age of 50 [4–6], and progresses to MM or related malignancy a rate of 1% per year [7, 8]. Smoldering multiple myeloma (SMM) is an intermediate asymptomatic condition that lies between MGUS and MM along the spectrum of clonal plasma cell proliferative disorders. It is important from a clinical standpoint to distinguish SMM from MGUS because the risk of progression of SMM is 10 times higher than MGUS in the first 5 years following diagnosis. SMM progresses to multiple myeloma at a rate of approximately 10% per year over the first 5 years following diagnosis, 3% per year over the next 5 years, and 1.5% per year thereafter [9]. Recent data confirm that the highest risk of progression is in the first 5 years, with 2-, 5-, and 10-year risk of progression of 22%, 42%, and 64%, respectively [10]. Thus, the type of follow-up and counseling are different for MGUS and SMM.

SMM is present in approximately 0.5% of the population above the age of 40 years [11], and accounts for approximately 15% of all cases of newly diagnosed MM [12–14]. It is distinguished from MGUS based on the M protein concentration and percentage of clonal BMPCs (Table 1) [15]. Light chain SMM is a unique subtype of SMM in which there is monoclonal free light chain (FLC) excess with no expression of an intact immunoglobulin heavy chain M protein such as IgG or IgA. It is characterized by the presence of ≥500 mg/24 h of monoclonal FLC on urine protein electrophoresis.

**Table 1:** International Myeloma Working Group Diagnostic Criteria for Multiple Myeloma and Related Plasma Cell Disorders.

Disorder	Disease Definition
IgM Monoclonal gammopathy of undetermined significance (IgM MGUS)	All 3 criteria must be met: • Serum IgM monoclonal protein <3 gm/dL • Bone marrow lymphoplasmacytic infiltration <10% • No evidence of anemia, constitutional symptoms, hyperviscosity, lymphadenopathy, or hepatosplenomegaly that can be attributed to the underlying lymphoproliferative disorder.
Non-IgM monoclonal gammopathy of undetermined significance (MGUS)	All 3 criteria must be met: • Serum monoclonal protein (non-IgM type) <3 gm/dL • Clonal bone marrow plasma cells <10%^a^ • Absence of end-organ damage such as hypercalcemia, renal insufficiency, anemia, and bone lesions (CRAB) that can be attributed to the plasma cell proliferative disorder
Light Chain MGUS	All criteria must be met: • Abnormal free light chain (FLC) ratio (<0.26 or >1.65) • Increased level of the appropriate involved light chain (increased kappa FLC in patients with ratio >1.65 and increased lambda FLC in patients with ratio <0.26) • No immunoglobulin heavy chain expression on immunofixation • Absence of end-organ damage that can be attributed to the plasma cell proliferative disorder • Clonal bone marrow plasma cells <10% • Urinary monoclonal protein <500 mg/24 h
Smoldering multiple myeloma	Both criteria must be met: • Serum monoclonal protein (IgG or IgA) ≥3 gm/dL, or urinary monoclonal protein ≥500 mg per 24 h and/or clonal bone marrow plasma cells 10–60% • Absence of myeloma defining events or amyloidosis
Multiple Myeloma	Both criteria must be met: • Clonal bone marrow plasma cells ≥10% or biopsy-proven bony or extramedullary plasmacytoma • Any one or more of the following myeloma defining events ◦ Evidence of end organ damage that can be attributed to the underlying plasma cell proliferative disorder, specifically: ▪ Hypercalcemia: serum calcium >0·25 mmol/L ( >1 mg/dL) higher than the upper limit of normal or >2·75 mmol/L ( >11 mg/dL) ▪ Renal insufficiency: creatinine clearance <40 mL per minute or serum creatinine >177 μmol/L ( >2 mg/dL) ▪ Anemia: hemoglobin value of >2 g/dL below the lower limit of normal, or a hemoglobin value <10 g/dL ▪ Bone lesions: one or more osteolytic lesions on skeletal radiography, computed tomography (CT), or positron emission tomography-CT (PET-CT) ◦ Clonal bone marrow plasma cell percentage ≥60% ◦ Involved: uninvolved serum free light chain (FLC) ratio ≥100 (involved free light chain level must be ≥100 mg/L and urine monoclonal protein level at least 200 mg per 24 h on urine protein electrophoresis) ◦ >1 focal lesions on magnetic resonance imaging (MRI) studies (at least 5 mm in size)

Modified from Rajkumar SV, Dimopoulos MA, Palumbo A, et al. International Myeloma Working Group updated criteria for the diagnosis of multiple myeloma. Lancet Oncol 2014;15:e538–e548.

^a^A bone marrow can be deferred in patients with low risk MGUS (IgG type, M protein <1.5 gm/dL, normal free light chain ratio), in patients with uncomplicated suspected IgM MGUS < 1.5 gm/dl, and in patients with light chain MGUS who have a serum FLC ratio <8, in whom there are no clinical features concerning for myeloma, macroglobulinemia, or amyloidosis.

Our recognition that the clinical course of SMM has a progression risk that decreases over time along with information from other laboratory studies has led us to better understand SMM as being a heterogenous clinically defined entity rather a than a true biologic intermediate stage between MGUS and MM. Thus SMM as currently defined includes some patients with biological pre-malignancy (biological MGUS) and some with biologic malignancy (multiple myeloma) [15, 16]. This is a major paradigm change, and over the last 10 years has initiated a cascading series of changes in our strategic approach to both SMM and MM. One of the initial goals was to immediately identify the group of SMM patients who have biologic malignancy that will declare itself with clinical complications within 2 years. Three biomarkers were validated: bone marrow clonal plasma cells ≥60%, serum involved to uninvolved free light chain (FLC) ratio ≥100 (provided involved FLC level is ≥100 mg/L), and more than 1 focal lesion (5 mm or more in size) on magnetic resonance imaging (MRI), each of which identified patients at high (approximately 80%) risk of progression within 2 years. These biomarkers were considered myeloma defining events (MDE), and adopted in 2014 in the International Myeloma Working Group (IMWG) Revised Diagnostic Criteria for multiple myeloma and related plasma cell disorders [15]. When using the FLC ratio ≥100 as a biomarker for the diagnosis of myeloma, it is important to ensure that the urinary monoclonal protein is concordant, with a level of at least 200 mg per 24 h on urine protein electrophoresis [17].

The revised IMWG criteria protect patients from end-organ damage and has eliminated a “catch-22” where we did not want end-organ damage but were also not willing to treat before end-organ damage occurred. It allowed therapy to be initiated before significant end-organ damage occurred. But it applied to a very small proportion of patients: the revised IMWG diagnostic criteria upstaged only about 10% of patients with SMM. The remaining patients are still considered SMM. Thus, although patients with imminent risk of progression were addressed by the 3 biomarkers considered MDEs in the revised IMWG diagnostic criteria, that still left approximately one-third of patients who already had malignant transformation to remain in the SMM category. These patients comprise most of the patients currently classified as high-risk SMM and account for the 50% or higher risk of progression to overt end-organ damage from MM within 2 years seen in this group. It is this category of patients that is currently the target population for early intervention.

## Clinical features

SMM is asymptomatic. It is recognized incidentally when patients are found to have a monoclonal protein during work up of a variety of different symptoms and laboratory abnormalities. In addition to the risk of progression to MM or AL amyloidosis, patients with SMM are also at risk of other systemic disorders besides overt malignancy that are causally associated with monoclonal proteins, including monoclonal gammopathy associated peripheral neuropathy and proliferative glomerulonephritis with immunoglobulin deposits [18, 19]. Similarly as with MGUS, there may also be an increased risk of venous and arterial thrombosis, infections, osteoporosis, and bone fractures even in the absence of progression to overt malignancy [20].

## Differential Diagnosis

SMM must be distinguished from MGUS and MM using the criteria listed in Table 1. Baseline laboratory studies should include complete blood count, serum creatinine, serum calcium, skeletal imaging with whole body low dose CT or positron emission tomography-computed tomography (PET-CT), serum protein electrophoresis (SPEP), serum immunofixation (IFE), 24 h urine protein electrophoresis (UPEP), urine IFE, and serum FLC assay [21]. An MRI of the spine and pelvis (or whole body MRI) should be considered in patients with suspected high risk SMM if skeletal imaging with CT or PET-CT is negative to ensure that focal myeloma defining lesions are not missed [22]. Bone marrow examination with fluorescent in situ hybridization (FISH) studies to detect high risk cytogenetic abnormalities (del 17p, t(4;14), gain 1q, del 13) and plasma cell immunophenotyping by multiparametric flow cytometry is needed.

## Prognosis

The risk of progression of SMM to MM or related malignancy is approximately 10% per year for the first 5 years, and then decreases over time. This rough estimate can be further refined using a variety of common variables, including the size and type of monoclonal protein, and the extent of bone marrow involvement [9]. For example, the time to progression was significantly shorter in patients with IgA M protein compared with IgG M protein, median 27 months versus 75 months, respectively, *P* = 0.004 [9]. It is not clear whether this difference is driven by isotype or underlying cytogenetic differences between the two groups. Similarly the time to progression was 117 months for patients with <20% bone marrow involvement versus 26 months for patients with bone marrow involvement by 20–50% clonal plasma cells, *P* < 0.001 [9]. A reduction in the level of uninvolved immunoglobulins is associated with increased risk of progression [9, 23]. The serum FLC ratio is also particularly valuable, and has been incorporated into risk stratification models [24].

Imaging studies are important for accurate diagnosis of SMM and specifically to exclude MM. They are also of value in identifying patients who do not meet criteria for MM but are nevertheless at higher risk of progression in the future. Thus patients with one focal non-osteolytic lesion and those with diffuse (non-focal) abnormalities on MRI are at increased risk of progression to MM, and require more close follow-up and repeat imaging in 3–6 months [25]. Similarly increased uptake on PET-CT without bone destruction is not adequate to be considered as an MDE; but is indicative of a higher risk of progression. Zamagni and colleagues found that the median time to progression was significantly shorter for patients with increased PET-CT uptake compared with patients with negative PET-CT, 1.1 years versus 4.5 years, *P* = 0.001. Progression occurred within 2 years in 58% of PET-CT positive patients versus 33% of PET-CT negative patients [26].

Bone marrow studies provide significant prognostic information beyond the extent of involvement. Immunophenotyping with multiparametric flow cytometry provides prognostic value by accurately distinguishing and quantitating bone marrow plasma cells with malignant potential (aberrant) from normal plasma cells [27]. In a Spanish study, the median time to progression was 34 months when bone marrow plasma cells were ≥95% aberrant versus not reached when bone marrow plasma cells had less than 95% aberrancy, *P* < 0.001. Detection of t(4;14) translocation, del(17p), and gain(1q) on FISH or other molecular studies is also associated with a higher risk of progression from MGUS or SMM to multiple myeloma [28–30].

Another important prognostic variable is change in one or more of the above parameters over time. In one study, an evolving change in monoclonal protein (0.5 gm/dl increase in M-protein) along with an evolving change in hemoglobin (0.5 g/dl decrease in hemoglobin) over a 12-month period was associated with high risk of progression [31]. Among patients with bone marrow plasma cells ≥20%, evolving M protein and evolving hemoglobin were independent predictors of progression; the 2-year progression rate was 90.5% in patients who had both an evolving M protein and evolving hemoglobin. The risk with evolving M protein been confirmed by an independent study by the Spanish myeloma group [32].

## Risk stratification

For clinical practice, the current goal of risk stratification is to identify patients with a 50% risk of progression within 2 years, since these are the patients who are most likely to already have biologic malignant transformation, and in clinical trials have shown the maximum benefit with early intervention. Multiple risk stratification models (eg. Spanish and Mayo Clinic models) have been proposed by combining prognostic factors [9, 23, 24, 28, 29, 33–36]. The Mayo 2018 criteria, also referred to as the 20-2-20 criteria, simplifies the identification of patients with high risk SMM using three variables: serum free light chain ratio >20, serum M protein level >2 gm/dL, bone marrow clonal plasma cells >20% [37]. The presence of 2 or 3 of these factors is associated with a median TTP to multiple myeloma of approximately 2 years, and is considered high risk SMM (Table 2). These criteria have been validated in a separate cohort by the IMWG [10]. The IMWG validation study also provides a scoring system for more accurate estimation of prognosis. Importantly, a recent study has found the Mayo 2018 high risk criteria also applies during follow-up when patients who are initially diagnosed as low risk SMM later evolve with higher M protein, serum FLC ratio, or bone marrow involvement. Such patients should be considered as newly diagnosed high risk SMM at that point and are candidates for clinical trials or early intervention.

**Table 2:** Risk stratification of smoldering multiple myeloma (SMM).

*Mayo 2018 Criteria (20-2-20 critieria)*
High risk SMM (2-year risk of progression 50%)
Any 2–3 of the following high risk factors:
Serum monoclonal protein > 2 gm/dL
Serum free light chain ratio (involved/uninvolved) >20
Bone marrow plasma cells >20%
Intermediate risk SMM
Any 1 high risk factor
Low risk SMM
No high risk factor
*International Myeloma Working Group Scoring System for SMM*^*a*^
High Risk SMM (2-year risk of progression, 75%)
Score >12
High-Intermediate Risk SMM (2-year risk of progression, 50%)
Score 9–12
Low-Intermediate Risk SMM (2-year risk of progression, 25%)
Score 5–8
Low Risk SMM (2-year risk of progression, 5%)
Score 1–4

^a^International Myeloma Working Group Scoring includes 4 components. Serum free light chain assay: Score of 0, 2, 3, and 5 for serum free light chain ratio 0–10, 11–25, 26–40, and >40, respectively; Serum protein electrophoresis: Score of 0, 3, and 4 for monoclonal protein level (gm/dL) 0–1.5, 1.6–2.9, and >3, respectively; Bone marrow: Score of 0, 2, 3, 5, and 6 for bone marrow plasma cell percentage 0–15, 16–20, 21–30, 31–40, and >40, respectively; Fluorescent in situ hybridization: Score of 2 for presence any high risk cytogenetic abnormality (del 17p, t(4;14), gain 1q, or del 13).

Derived from Lakshman A, et al. Risk stratification of smoldering multiple myeloma incorporating revised IMWG diagnostic criteria. Blood Cancer J 2018;8:59; and Mateos MV, et al. International Myeloma Working Group risk stratification model for smoldering multiple myeloma (SMM). Blood Cancer J 2020;10:102.

## Treatment

**Fig. 1:**
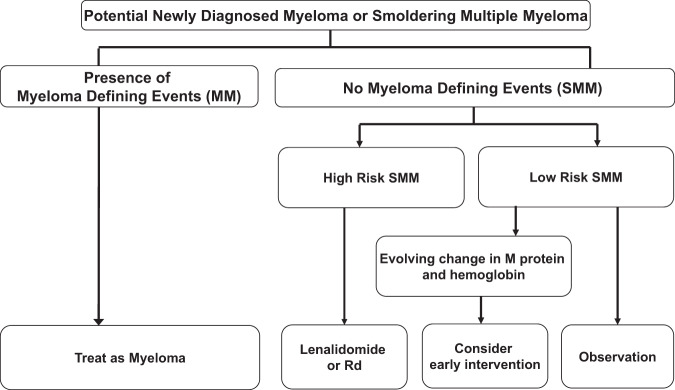
Approach to the management of smoldering multiple myeloma. Footnote for Fig. 1: SMM, smoldering multiple myeloma; MM, multiple myeloma; Rd, lenalidomide plus dexamethasone. Myeloma Defining Events: End organ damage felt to be related to myeloma (hypercalcemia, light chain cast nephropathy, anemia, osteolytic bone lesions), serum free light chain ratio ≥100 with involved serum free light chain level ≥100 mg/dL and urine monoclonal protein ≥200 mg per 24 h on urine protein electrophoresis, ≥60% clonal bone marrow plasma cells, >1 focal lesion on magnetic resonance imaging. High risk Smoldering Multiple Myeloma: Any 2 of the following: bone marrow plasma cells >20%, serum monoclonal protein >2 gm/dL, serum free light chain ratio >20. Or high risk score based on the International Myeloma Working Group Scoring System for Smoldering Multiple Myeloma. Evolving change: Increase in monoclonal protein of 0.5 gm/dl or more along with a concomitant decrease in hemoglobin of 0.5 g/dl or more over a 12-month period.

Our current approach to management of SMM is provided in Fig. 1 [38]. For patients with low risk SMM by the 20-2-20 criteria, observation remains the standard of care. In these patients, serum M protein, serum FLC levels, complete blood count, serum calcium, and serum creatinine should be monitored every 3–4 months. The interval for follow-up can be reduced to once every 6 months after the first 5 years [38]. If during follow-up, low risk SMM patients with 20% or greater bone marrow involvement develop an evolving change in monoclonal protein level accompanied by an evolving change in hemoglobin (as discussed earlier), treatment should be considered. These recommendations are based on data showing that such increase is associated with >90% risk of progression within 2 years [31]. In patients with MRI showing diffuse infiltration, solitary focal lesion, or equivocal lesions, follow-up radiographic examination in 3–6 months is recommended [25]. During follow-up, if low risk SMM patients meet criteria for high risk SMM based on the Mayo 2018 or IMWG risk stratification model, early intervention similar to high risk SMM described below should be considered.

For patients with newly diagnosed high risk SMM, we recommend therapy with lenalidomide or lenalidomide plus dexamethasone (Rd) for two years, or enrollment in a clinical trial testing early therapy. Early studies in SMM with alkylating agents found no significant benefit [39–41]. A subsequent randomized trial comparing thalidomide plus zoledronic acid versus zoledronic acid alone in patients with SMM showed some promise [42]. Time to monoclonal protein elevation was superior for patients treated with thalidomide plus zoledronic acid (*n* = 35) versus zoledronic acid alone (*n* = 33). However, there were no significant differences in time to end organ damage, 4.3 versus 3.3 years, and no difference in overall survival, 5-year survival 74% versus 73%, respectively. Further, thalidomide has long-term side effects that make not suitable for treatment of SMM [43, 44]. These early trials were also limited by lack of a risk-adapted strategy.

Two randomized trials with lenalidomide in high risk SMM have shown benefit. In the Spanish randomized trial in patients with high risk SMM, time to progression to MM with end organ damage was significantly longer in patients treated with Rd compared with observation, median TTP not reached versus 21 months, *P* < 0.001 [27, 45]. Overall survival was also longer, 3-year survival rate 94% vs. 80%, respectively, *P* = 0.03. Importantly, early intervention with Rd did not affect the impact of subsequent therapy after progression or survival after progression, arguing against any long-term deleterious effect of early intervention. More recently a randomized trial conducted by the Eastern Cooperative Oncology Group (ECOG) found that early therapy with lenalidomide as a single agent prolongs time to symptomatic MM with end-organ damage in patients with high risk SMM [46]. Only 6 patients have died in this trial, 4 in the observation arm and 2 in the lenalidomide arm, making it difficult to assess the effect of early therapy on overall survival. However, when the effect of early therapy is analyzed among patients meeting Mayo 2018 high risk criteria, both the Spanish trial and the ECOG trial show a striking 90% reduction in time to end-organ damage. Based on the results of these two trials, we recommend that patients with newly diagnosed high risk SMM patients be considered for early intervention with lenalidomide or Rd for two years (Fig. 1). Between lenalidomide and Rd, the choice should be made taking into account the patients age, comorbidities, and tolerance to dexamethasone. Patients with high risk SMM who are treated with lenalidomide or Rd should have peripheral blood stem cells collected for cryopreservation after approximately 4–6 cycles of therapy [47, 48]. Patients with high risk SMM are also candidates for clinical trials testing intensive therapy with curative intent [49]. Our recommendation in favor of early intervention applies to patients recently diagnosed with high risk SMM. We recognize that there are patients with high risk SMM who have been diagnosed years ago and have remained stable without therapy. These patients represent a self-selected group with likely stable pre-malignancy and can therefore continue to be observed closely and considered for intervention only at time of evolving laboratory parameters.

The role of bisphosphonates to delay bone events in SMM is not fully settled. In a randomized trial, a reduction in skeletal-related events (SRE) has been seen with pamidronate (once a month for 12 months) compared with observation [50]. However, no improvement in time to progression or survival was seen. In another randomized trial, a reduction in SREs was noted with zoledronic acid (once a month for 12 months), 56% versus 78%, respectively, *P* = 0.04 [51]. We recommend once-yearly bisphosphonate similar to that used for the treatment of osteoporosis for patients with SMM who have osteopenia or osteoporosis.

## Common questions and controversies

### Can we remove the SMM category and merge it with MGUS or MM?

Although biologically, there is only a clear distinction between MGUS (pre-malignancy, analogous to a polyp) and MM (malignancy), for clinical purposes SMM is an important entity to preserve. It is easy to distinguish from MGUS, SMM, and MM with current clinical criteria, and the distinctions have major clinical implications for the patient in terms of prognosis, management, and for planning their life. A puristic focus based on biology does not help in the clinic. Importantly, the diagnosis of SMM highlights the increased risk of progression and the need for closer follow-up compared to MGUS, a distinction that will be lost if we clubbed the two together.

The SMM category is similar to staging systems based on tumor volume, nodal spread, and cellular characteristics used in solid tumors. They may not be biologically different, but the clinical implications are different. In the plasma cell disorders field, we did once refer to SMM as Stage I MM. But since the SMM terminology has been in use for decades, we feel no reason to rename it at this point. If we come to a point where accurate tests to classify patients with SMM into those with biologic pre-malignancy versus biologic malignancy are validated and widely available, and when we have data from randomized trials that treating high risk SMM similar to MM provides superior clinical benefit compared to lenalidomide or Rd, we can reconsider. We are not there yet, and we will not be there for a while.

### Are the current risk stratification models adequate?

Current risk stratification models are not perfect, but they are readily available around the world, and identify patients with a 50% risk of progression within 2 years. The population so identified has been treated in randomized trials with early intervention, and a 90% reduction in risk of end organ damage has been demonstrated in both trials, with a survival advantage in one trial. It is possible to do better and we will continue to develop better models. Secondly, the fact that different models capture different high-risk populations is not a negative. Each of our models is not very sensitive, and so we capture only a proportion of patients at risk. By using more models, we capture more of the patients at risk, and lack of an overlap is actually advantageous in this regard. But whatever model is used, as long as the population identified has at least a 50% risk of progression in 2 years, it is sufficient for clinical purposes both for management and counseling. Third, modern genomic sequencing methods have not shown clear superiority in identifying patients for early intervention compared to more conventional risk stratification models. Further, they are not standardized, with methods and techniques varying across laboratories, and are not widely available. When available, we encourage their use in addition but not to the exclusion of existing systems. In the future, assessment of circulating tumor cells is another emerging technology that can be standardized and serve as a widely available metric for risk stratification and follow-up.

### Is early therapy justified without clear overall survival benefit?

In MM, end-organ damage includes osteolytic bone lesions and renal failure. At times, these are not reversible. They can cause significant morbidity to patients. We feel reducing risk of bone lesions and renal failure is sufficient clinical benefit to justify the intervention. If a decision is made not to offer treatment to patients with newly diagnosed high risk SMM, patients should be advised that two trials have shown 90% reduction in end organ damage along with a clear discussion on the pros and cons. Further, in many parts of the world lenalidomide is inexpensive and cost is not a barrier to initiation of therapy.

### Why can we not observe patients closely for progression instead of starting on any form of therapy?

In our experience conducting retrospective and prospective studies on hundreds of patients with SMM [9, 10, 27, 31, 37, 42, 52, 53], we have seen physicians reassure patients with high risk SMM that the disease is stable only to have progression occur in between visits. In randomized trials conducted in specialized centers we note 90% reduction in end organ damage with simple lenalidomide or Rd therapy compared with observation alone. Thus, attempting to delay therapy until the last minute and intervene in time before end organ damage occurs is easier said than done and had not been shown to be possible in clinical trials even with monthly follow-up.

### Why do we recommend lenalidomide or lenalidomide plus dexamethasone instead of myeloma-like therapy for high risk SMM?

We have data from randomized trials that lenalidomide or Rd is superior to observation in preventing end organ damage [45, 52]. One of these trials has shown a clear overall survival benefit [45]. Although it is interesting to hypothesize that a myeloma-like triplet or quadruplet regimen may be superior to lenalidomide or Rd, we do not have randomized data to support that. Such a trial is ongoing (NCT03937635), and we are awaiting its results. For those concerned that lenalidomide or Rd may cause some delayed harm or drug resistance, that experiment has been done: the recent two randomized trials with lenalidomide do not show any such adverse effect [45, 52]. This is therefore a theoretical risk that has been tested and found to be not true. For those who feel we should go straight to myeloma-like therapy it is also worth considering that most patients cannot access such therapy without regulatory approval. In a disease where observation has been the standard of care, we need to first demonstrate one drug works compared to no treatment, and then build on that. Making a practice change without proof will provide myeloma therapy to well insured patients in the United States but leave the vast majority in the world with no approved intervention. We are doing several clinical trial strategies in parallel including necessary regulatory trials (to show one drug works versus observation)(NCT03301220), strategic trials (to see if myeloma like therapy is superior to Rd)(NCT03937635), and more aggressive trials (to see if early aggressive intervention at the SMM stage can be curative)(NCT02415413, NCT03289299).

## Future directions

An ongoing ECOG randomized trial is testing whether a standard myeloma therapeutic triplet (DRd) will be superior to prophylactic doublet therapy with lenalidomide plus dexamethasone in patients with high risk SMM (NCT03937635) [54]. A similar randomized trial is comparing isatuximab plus Rd versus Rd in patients with high risk SMM (NCT04270409). There are also clinical trials testing intensive therapy with curative intent [49].

## Data Availability

This is a current treatment algorithm, There are no new data generated for this manuscript and data sharing is not applicable.
